# Comparative and Meta-Analysis Evaluation of Non-Destructive Testing Methods for Strength Assessment of Cemented Paste Backfill: Implications for Sustainable Pavement and Concrete Materials

**DOI:** 10.3390/ma18122888

**Published:** 2025-06-18

**Authors:** Sakariyau Babatunde Abdulkadir, Qiusong Chen, Erol Yilmaz, Daolin Wang

**Affiliations:** 1 School of Resources and Safety Engineering, Central South University, Changsha 410083, China; skymorbabs@csu.edu.cn (S.B.A.); daolinw@csu.edu.cn (D.W.); 2 Department of Resources Engineering, University of Science and Technology Beijing, Beijing 100083, China; 3 Department of Civil Engineering, Geotechnical Division, Recep Tayyip Erdogan University, Fener, Rize TR53100, Türkiye; erol.yilmaz@erdogan.edu.tr

**Keywords:** cemented paste backfill, non-destructive testing (NDT), strength prediction, sustainability in mining, ultrasonic pulse velocity (UPV)

## Abstract

Cemented paste backfill (CPB) plays an important role in sustainable mining by providing structural support and reducing surface subsidence. While traditional destructive testing methods such as unconfined compressive strength (UCS) tests offer valuable understanding of material strength, they require a lot of resources, are time-consuming, and environmentally unfriendly. However, non-destructive testing (NDT) techniques such as ultrasonic pulse velocity (UPV), electrical resistivity (ER), and acoustic emission (AE) provide sustainable alternatives by preserving sample integrity, minimizing waste, and enabling real-time monitoring. This study systematically reviews and quantitatively compares the effectiveness of UPV, ER, and AE in predicting the strength of CPB. Meta-analysis of 30 peer-reviewed studies reveals that UPV and AE provide the most consistent and reliable correlations with UCS, with R^2^ values of 0.895 and 0.896, respectively, while ER shows more variability due to its sensitivity to environmental factors. Additionally, a synthetic model combining UPV, AE and ER demonstrates improved accuracy in predicting strength. This hybrid approach enhances predictions of material performance while supporting sustainability in mining and construction. Our research advocates for better testing practices and presents a promising direction for future infrastructure projects, where real-time, non-invasive monitoring can enhance material performance evaluation and optimize resource use.

## 1. Introduction

Cemented paste backfill (CPB) is a modern approach applied in sustainable mining practices, especially in the underground mines, to address the problems associated with mine tailings and to provide structural support for roadways or pavement materials. CPB is a composite material to fill voids, which also helps mitigate surface subsidence and improve ground conditions [1,2,3]. CPB must meet specific compressive strength requirements to ensure structural stability. Industry standards ASTM C39/C39M-24 [4] and practical guidelines mandate minimum strengths of >0.7 MPa for temporary structures and >1.5 MPa for long-term stability [5]. These thresholds ensure safe ground support and prevent subsidence risks.

The strength of CPB, pavement, and other concrete materials is often tested by methods that involve breaking the material samples. A well-known test for this is the unconfined compressive strength (UCS) test. Even though this test provides important information, it requires a lot of resources, produces waste, and takes a lot of time to complete. These drawbacks make it not very suitable for practices that aim to be environmentally friendly and sustainable [6,7]. Such limitations become particularly problematic when assessing backfill performance in active mining areas where real-time strength evaluation is crucial for safety.

As the mining industry strives to be more environmentally friendly, finding ways to check materials without causing damage is crucial. Methods such as ultrasonic pulse velocity (UPV), electrical resistivity (ER), and acoustic emission (AE) offer non-destructive solutions [8,9]. These techniques help maintain the integrity of samples, cut down on waste, and provide real-time insights into material properties. This shift promotes resource efficiency and helps us understand their sustainability, durability and strength prediction in various construction applications [10,11]. Although this study focuses on CPB, the NDT methods discussed, such as UPV, ER and AE are also widely applicable in assessing pavement materials, concrete and asphalt.

The properties of CPB have usually been assessed through destructive testing methods, such as UCS, triaxial tests, and tensile strength tests. While these methods provide accurate and reliable results, they are inherently limited by their destructive nature [12,13,14]. Destructive testing entails pulling out and destroying samples, which not only undermines the integrity of the backfill structure but also restricts the scalability and frequency of testing [15,16,17]. Furthermore, these methods are time consuming, labor-demanding and not very suitable for use in real time conditions [18,19].

Recent progress in NDT methods such as UPV, ER, and AE holds promise for CPB assessment. UPV is used to measure how quickly waves travel through materials, providing information about their density and elasticity [15,16]; ER examines electrical conductivity, which helps assess hydration levels and porosity [18]; and AE identifies tiny cracks that form under stress, offering early warnings of potential failures [19,20]. Individually, these methods address specific DT shortcomings: they preserve sample integrity, enable real-time monitoring, and reduce costs by up to 40% [21,22]. However, existing studies remain fragmented, with no systematic comparison of their reliability across diverse CPB compositions or environmental conditions [23,24]. For instance, while Yılmaz and Ercikdi [25] demonstrated UPV’s strong UCS correlation (R^2^ = 0.79–0.95) in silica-rich tailings, Wu et al. [26] noted ER’s susceptibility to moisture fluctuations—a gap this review bridges through meta-analysis.

This paper presents a systematic review of how three important NDT methods like UPV, ER, and AE perform in predicting the strength of CPB focusing on their sustainability aspects. It highlights the benefits of these methods, focusing on how they can be better for the environment and help reduce costs for the mining and construction sectors. We quantify the predictive power of UPV, ER, and AE for UCS through meta-analysis, elucidating method-specific strengths and limitations using 30 relevant studies.

## 2. Testing Methods Overview

**Figure 1:**
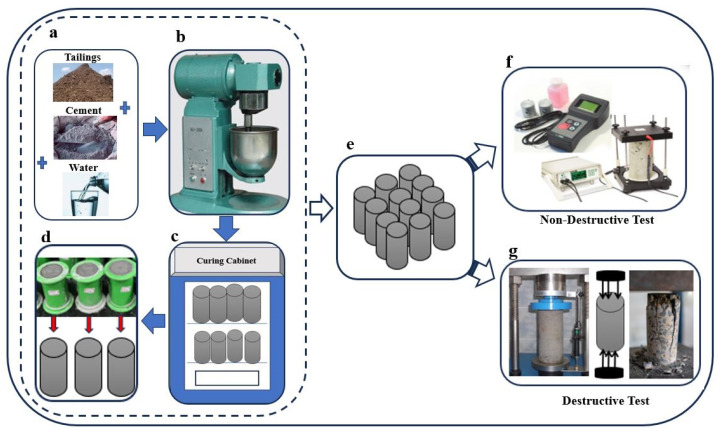
Presents (**a**–**d**) Step by Step CPB preparation, (**e**) required sample for testing process, (**f**) non-destructive methods and (**g**) destructive testing method.

Figure 1 presents the sequence of steps followed in the CPB process to prepare the samples and subjected them to destructive and non-destructive assessment. In step (a), the raw materials—cement, water and tailings—are collected. In step (b), these substances are put into an automatic mixer to create a uniform paste mixture. Step (c) entails pouring the mix that has been prepared into cylindrical molds to make sure that it is uniform and compacted to the right level. The samples are then cured in a controlled surrounding (d) using a curing machine to provide the right temperature and humidity conditions. After curing (e), the hardened CPB samples will either or both undergo (f) NDT and (g) DT for mechanical assessments to determine their structural integrity and performance.

### 2.1. Overview of Destructive Testing Methods

Destructive testing (DT) methods are necessary for identifying the mechanical behavior of materials especially in construction and engineering fields. The approaches entail the use of forces on the materials up to failure, to determine important characteristics such as the load carrying capacity, toughness and the general performance. This methodology is well established and conforms with industry standards for the evaluation of structural materials and provides concise and quantifiable information on their performance during stressful conditions [27,28,29].

Among the DT methods, the UCS test is one of the most common for CPB, where a cylindrical sample of CPB is subjected to axial compression up to failure, and the stress–strain relationship is recorded to determine the maximum compressive stress that the material can tolerate [30,31,32]. This parameter is very important in determining the load carrying capacity and the structural integrity of CPB and therefore affects the safety and efficiency of mining operations. UCS test results are used in the optimization of CPB mix designs to ensure that the mixes developed are capable of meeting the required strength criteria for the various applications especially in underground mining where it is essential that the CPB has enough strength to support the surrounding rock masses [17,33].

Another important method of destructive testing is the triaxial test that receives mechanical properties of CPB under in situ-like conditions. This test applies a controlled stress to a cylindrical sample in three orthogonal directions, so as to determine shear strength and failure mechanisms. Complex loading conditions of mining environment are simulated by triaxial tests made on CPB to determine its behavior. While most CPB testing focuses on static loads, dynamic load studies like drop-weight or split-Hopkinson bar tests are emerging to simulate blast or seismic impacts [34,35]. These remain less common due to CPB’s primary role in static load-bearing. These tests outcomes reveal that with confining pressure, both peak and residual strengths rise, vital information for optimizing mix designs for geological stress resistance [36]. Triaxial tests are nevertheless costly and time consuming because they require specialized equipment and very precise sample preparation.

The Brazilian test, also known as the splitting tensile strength test, is another method employed to measure the tensile strength of brittle materials such as CPB. This test induces tensile stresses that lead to failure along the vertical plane by applying a compressive load diametrically across a cylindrical sample [37,38]. This test is especially significant for determining CPB resistance to cracking and failure that is critical for maintaining the stability and durability of structures in mining operations. However, like other destructive tests, it is limited in its ability to allow samples to be used for further analysis and may not accurately replicate field conditions because of differences in stress distribution [39,40,41].

Table 1 below highlights the fundamental differences between these three commons destructive (UCS, Triaxial and Brazilian) testing methods used to evaluate the mechanical properties of CPB and similar materials. The UCS test measures uniaxial compressive strength under zero confinement, providing a baseline for material performance. The Triaxial test incorporates confining pressure to simulate in situ stress conditions, yielding shear strength parameters critical for geotechnical applications. In contrast, the Brazilian test indirectly assesses tensile strength, a key property for predicting crack initiation in brittle CPB structures.

**Figure 2:**
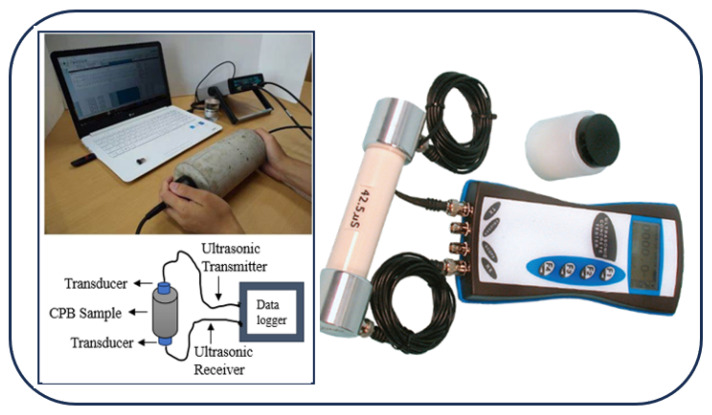
Image showing UPV Testing Setup as NDT method for CPB sample. It includes an ultrasound transmitter, a transducer for the CPB sample, a data logger, and an ultrasound receiver.

Destructive testing methods provide valuable insights into the mechanical properties of CPB, but they suffer from inherent limitations, including sample destruction, time-consuming preparation, labor intensity, and limited scalability. Additionally, these methods cannot simulate real-world conditions, lack repeatability, require complex data interpretation and generate material waste [42,43]. As summarized in Table 1, these drawbacks have motivated researchers to adopt NDT methods such as UPV and ER testing as visualized in Figure 1 and Figure 2 which preserve sample integrity while offering real-time, scalable, and repeatable assessments of CPB properties without compromising structural capacity.

### 2.2. Non-Destructive Testing Methods

- Ultrasonic Pulse Velocity (UPV) Testing

UPV testing is a non-destructive testing method that is applied widely for the assessment of the physical properties of materials such as CPB. It works by sending high frequency ultrasonic waves into a material and then measuring the velocity of the waves. The wave velocity is a function of material density, elasticity and compressive strength and thus enables the engineer to make property evaluations without having to destroy the sample. It gives information about the internal structure and integrity of CPB by analyzing the time taken by the waves to travel through the material and any changes in the amplitude or frequency of the waves [44,45].

The UPV testing setup, which is illustrated by Figure 2, utilizes an ultrasonic transmitter and a transducer that is responsible for generating and sending ultrasonic waves through the material under examination is referred to as the setup. These signals are sent through the material and are received by ultrasonic cables which are connected to a data display and logger unit for analysis. The facts about the integrity, homogeneity and possible flaws of the substance are obtained from the captured data [46,47]. This method is especially useful for quality control and monitoring in both laboratory and field conditions since it allows multiple evaluations of the same specimen or structure at different times [48,49].

UPV presents information online, which is very useful in making on-the-spot decisions and changes during construction or mining operations [50]. For these reasons, it is a suitable tool for examining the CPB at different levels, from material testing at the initial stage to monitoring during the service period. However, the precision of the results depends significantly on proper calibration, which needs reference samples with known properties. Another issue is that material heterogeneity, such as density variations, voids, and cracks, can influence the propagation of ultrasonic waves, resulting in irregular outcomes [51,52,53].

- Electrical Resistivity Testing

The principle of electrical resistivity testing is based on the fact that the resistivity of a material is a function of its physical properties. Thus, porosity and microstructure of the cement paste changes with the degree of hydration, which, in turn, affects the electrical resistivity. Lower resistivity is a characteristic of porous structures while higher resistivity is observed in more dense and better hydrated materials [54,55]. The setup for resistivity measurements is simple and can be used in the field and the laboratory. This method is also suitable for online control that enables online assessment of the curing process and the quality of CPB. This correlation highlights the importance of resistivity testing as an early warning tool in assessing the performance of CPB [56,57,58].

However, there are some demerits of using electrical resistivity testing. A major drawback is the sensitivity to moisture content; changes in moisture can lead to changes in resistivity which can make data analysis difficult [59]. The moisture content of CPB can be a major factor in the electrical properties of the mixture, and thus, proper control and measurement of the environmental conditions during testing is very essential [60]. Moreover, the analysis of resistivity data is quite complex and requires knowledge of the materials structure and hydration mechanism since resistivity by itself is not a full characterization of the material state [61,62].

- Acoustic Emission (AE)

The AE test is based on the detection of transient elastic waves emitted when energy is released from a material containing localized sources of energy such as microcracks and fractures [63,64]. CPB will be subjected to stress and the micro events generate acoustic signals that can be caught by sensitive sensors. The information derived from the analysis of these signals gives information on the damage mechanisms occurring inside the backfill material and the growth of cracks [65,66]. The AE is suitable for monitoring damage evolution in CPB and real time assessment of its stability is possible [67].

A major strength of AE is that it can provide real time monitoring of the propagation of cracks and failure mechanisms, which is very useful in the study of CPB behavior under load and failure prediction [27,68]. Moreover, AE can be employed for the discrimination of the initial hydration products in cement composites and therefore to determine the strength and durability of CPB [69]. Furthermore, AE is suitable for continuous monitoring of large areas, which is important for numerous applications of backfill in mining, where the conditions are usually dynamic [70].

However, there are some significant demerits of AE which cannot be ignored. The major restriction is that accurate capturing and analysis of acoustic signals need sensitive and high-quality equipment, and noise can be a problem [64]. It is important to separate the meaningful acoustic signals from the noise for accurate evaluation. Moreover, AE data analysis can be rather complicated, and the signals are not always straightforward to link to certain damage types. Therefore, the analysis of the data and the conclusions about the state of CPB made from it requires professional knowledge [71,72,73].

## 3. Methodology: Systematic Review and Meta-Analysis Protocol

### 3.1. Literature Search Strategy

**Figure 3:**
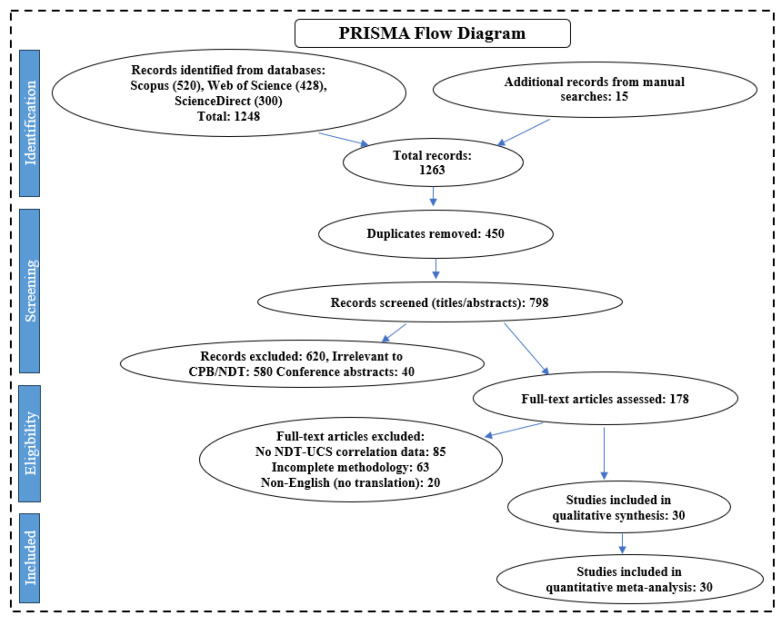
PRISMA flowchart illustrating the method used to search and select literature systematically. From the 1248 records identified, 30 studies were chosen for the meta-analysis.

We explored five major research databases such as Scopus and Web of Science, depicted in Figure 3. Our search included terms like “cemented paste backfills” or “CPB”, “non-destructive testing” or “NDT”, as well as “ultrasonic”, “electrical resistivity”, “acoustic emission”, and “strength prediction” or “UCS correlation”. We focused on peer-reviewed studies from 2010 to 2025 that demonstrated clear quantitative links between NDT techniques like UPV, ER, or AE, and UCS. These studies needed to detail their CPB mix designs, including specifics about binder types and tailings compositions. They also had to provide statistical data, such as R^2^ data, to calculate effect sizes. We excluded studies solely using DT methods, focusing on non-cemented backfills, duplicates, conference summaries, or those lacking comprehensive methodological details.

### 3.2. Study Screening and Statistical Analysis

The PRISMA framework, as shown in Figure 3, was selected for its rigor in systematic reviews, minimizing selection bias via transparent screening stages. Initially, we identified 1248 records and eliminated 450 duplicates. Subsequently, 620 studies were dismissed, as their titles and abstracts did not pertain to CPB/NDT. Next, we assessed 178 studies in full to determine their relevance. Out of these, only 30 met all criteria. We prioritized studies with controlled experimental conditions, such as ASTM/ISO compliant curing protocols, included at least three samples per mix design, and provided clear error metrics like standard deviations. This thorough process ensured we included high-quality, reproducible data for meta-analysis using Python 3.9 (libraries: NumPy, pandas, scikit-learn, and Matplotlib) to assess data consistency through R^2^ values, and create forest plots to evaluate different NDT methods.

## 4. Comparison of Destructive and Non-Destructive Testing

**Figure 4:**
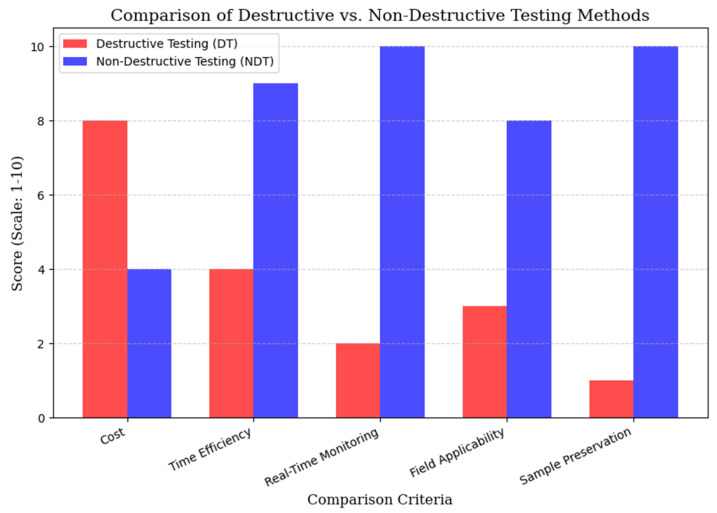
Bar chart comparing DT and NDT across key assessment criteria. It focuses on important factors like saving resources, reducing costs, and material integrity preservation.

Table 2 and Figure 4 compare DT and NDT methods using qualitative scores (1–10 scale). While DT provides highly accurate strength data through destructive failure testing, it suffers from sample destruction, high costs, specialized equipment requirements, and slow turnaround—limiting its field applicability. In contrast, NDT techniques preserve sample integrity, enabling real-time monitoring and repeat assessments with moderate-to-high precision. Their cost-effectiveness, efficiency, and field adaptability make NDT particularly valuable for large-scale applications across mining, construction, and manufacturing sectors, where operational continuity is crucial. The quantitative comparison highlights NDT’s advantages in sustainability and scalability while acknowledging DT’s superior absolute accuracy for controlled laboratory conditions.

### 4.1. Case Studies and Applications

Various NDT methods are used to assess the properties of CPB in both laboratory and field conditions. The following are 10 case studies for each NDT method, along with their effectiveness, challenges and implications for CPB assessment.

#### 4.1.1. Ultrasonic Testing Method

**Figure 5:**
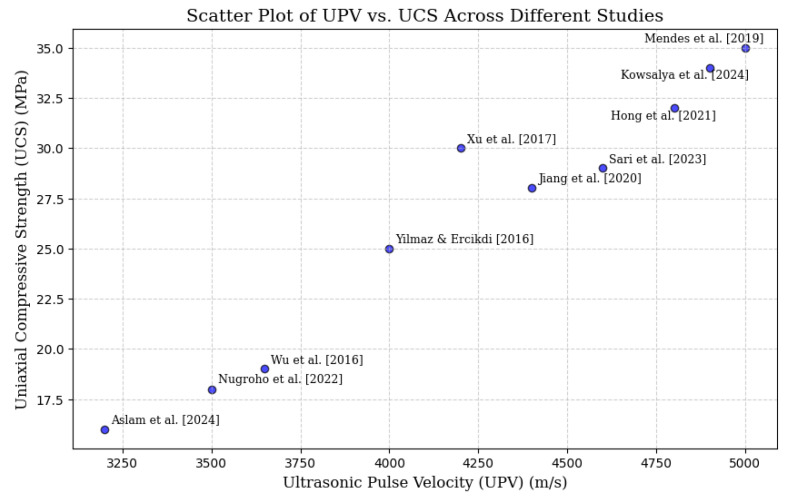
Scatter plot graph showing the relationship between UPV and UCS across different studies [13,16,24,53,74,75,76,77,78,79].

**Figure 6:**
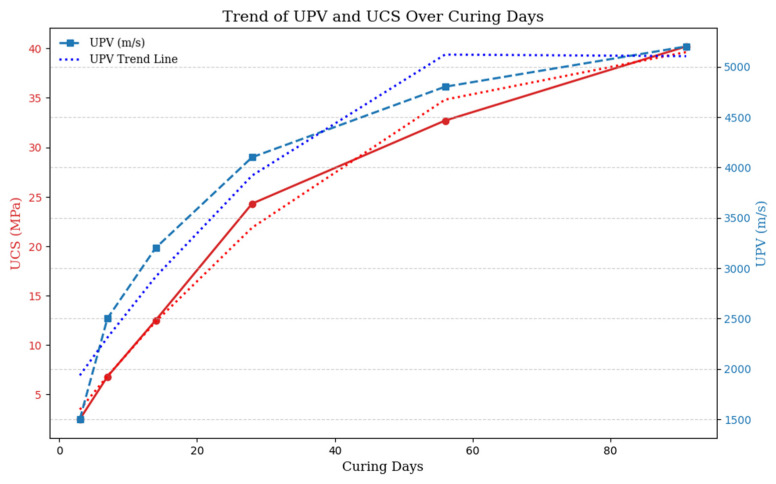
This figure shows the evolution of UCS (MPa) and UPV (m/s) with curing days. The red dotted line represents the polynomial trend for UCS, while quadratic trend lines (dashed) are fitted for both UCS and UPV, highlighting their increase over time.

To enhance clarity and enable rapid visual comparison, Figure 5 and Figure 6 present the correlation studies between UPV and UCS from Table 3 in the form of scatter plots and trend graphs. This visual representation effectively illustrates the relationship between UPV and UCS across multiple studies, thus enabling an at-a-glance comparison of the NDT systems.

Several research studies presented in Table 3 show that UPV can be used as a reliable, non-destructive alternative to UCS for the evaluation of cemented materials, concrete and paste backfill. Studies have revealed UPV-UCS correlations across curing periods and material compositions, confirming UPV ability to predict strength in CPB, cemented gangue backfill (CGB), and alkali-activated slag-based backfill [74,75,76]. It has been highlighted that UPV’s utility in assessing concrete quality, especially in silica fume (SF) and varied binder mixtures. In addition, studies have further validated UPV for sustainable lightweight and fly ash-based concretes by monitoring hydration, density, and porosity, respectively [77,78]. Therefore, UPV can provide a non-invasive and efficient real-time estimation of UCS for improving quality control in mining and construction.

The studies presented in Table 3 collectively define UPV as a strong candidate for predicting UCS in a variety of cementitious materials, such as CPB, gangue backfill (CGB), alkali activated slag based backfill, and concrete mixtures. The studies show high correlation coefficients (R^2^ > 0.85), which prove that UPV can be used to monitor the hydration process, material density and porosity. The study on sustainable materials (such as silica fume modified and lightweight concrete) also confirms the suitability of UPV for assessing different binders and curing conditions. As a non-destructive and online measurement method, UPV is shown to be applicable for practical use as an alternative to UCS testing in mining, construction, and material engineering.

#### 4.1.2. Electrical Resistivity (ER) Testing

**Figure 7:**
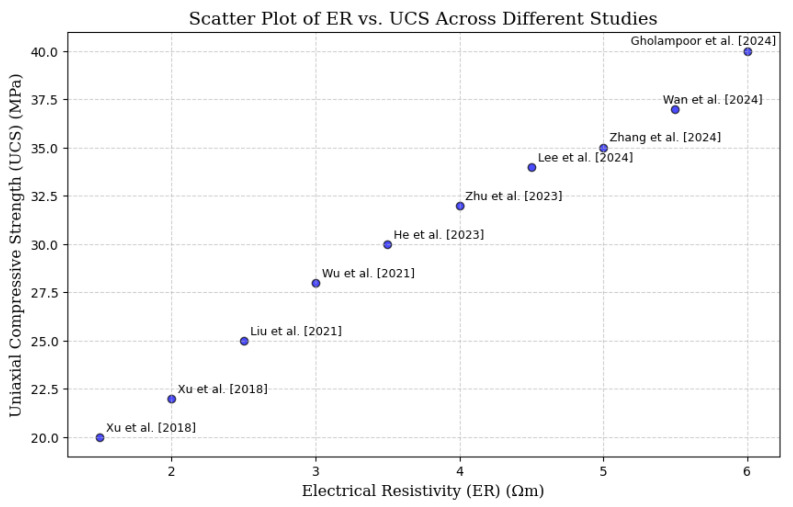
Scatter plot graph showing the relationship between ER and UCS across different studies [25,60,80,81,82,83,84,85,86,87].

**Figure 8:**
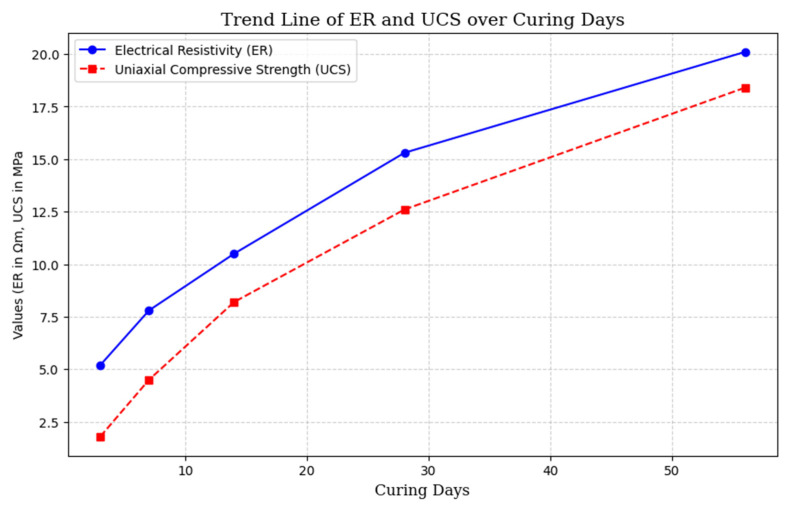
Present trend line showing how ER and UCS change over curing periods.

To improve readability and enable fast visualization of the data, Figure 7 and Figure 8 present the correlation studies between ER and UCS from Table 4 in a scatter plot with a trend line. This graphical representation effectively shows the connection between ER and UCS across multiple studies, facilitating a more straightforward comparison of the non-destructive testing (NDT) systems.

The Correlation Studies in Table 4 have proven ER to be a non-destructive alternative to UCS testing to determine the mechanical properties of CPB, concrete and other cementitious products. Figure 7 and Figure 8 visualize a good correlation between ER and UCS and that ER can be used to monitor hydration, porosity and microstructural evolution during curing periods. However, sample non-homogeneity, particularly in the case of air voids or uneven binder distributions, can significantly affect ER measurements by disrupting ionic conduction pathways, potentially leading to localized resistivity variations of up to 20% [86,87,88]. The results show that higher ER values are associated with higher UCS, and this is because of material densification and reduced permeability. Further work on hydration mechanisms, curing temperatures, and additives like calcium nitrite and slag also corroborates ER predictive role. As ER can monitor strength evolution in real-time without destroying specimens it emerges as a low-cost and time-saving alternative to UCS and finds its place in quality control in mining, geotechnical engineering and construction.

#### 4.1.3. Acoustic Emission (AE) Monitoring

**Figure 9:**
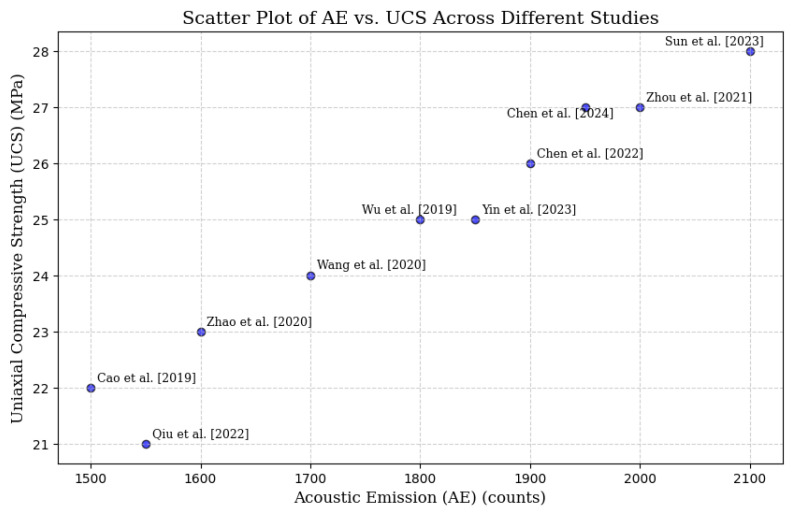
Scatter plot graph showing the relationship between AE and UCS across different studies [88,89,90,91,92,93,94,95,96,97].

**Figure 10:**
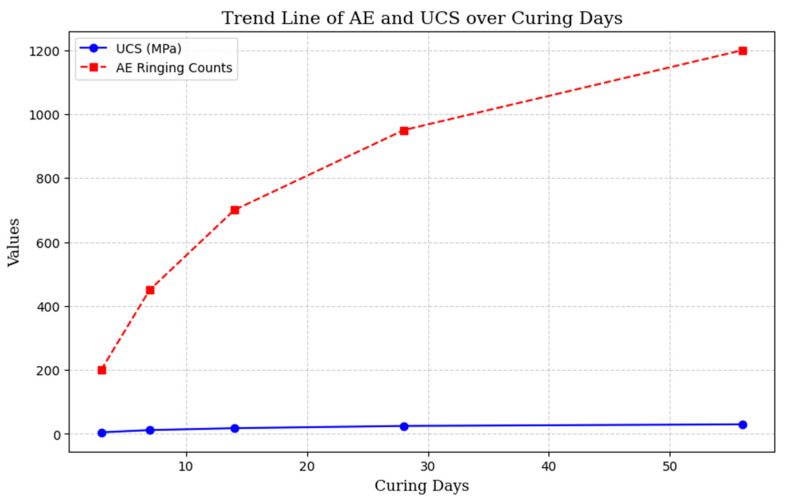
Present trend line showing how AE and UCS change over curing periods.

To enhance understanding and allow for quick data observation, Figure 9 and Figure 10 depict the correlation between AE and UCS, using data from Table 5. These figures include a scatter plot showing the relationship between AE and UCS across different studies and a trend line that clearly illustrates the relationships found in various studies. This visual format makes it easier to compare non-destructive testing (NDT) systems.

AE monitoring has been established as a reliable non-destructive alternative to the conventional UCS testing for determining the mechanical properties and failure behavior of CPB and other related materials. The AE parameters such as ring count and strength rate have a good correlation with stress–strain conduct and can capture the crack growth and failure process [88,89]. In addition, the AE is capable of monitoring real-time crack propagation and changes in structural integrity under different stress conditions [90,91]. Thus, AE can be used to assess the mechanical properties and failure behavior of CPB.

Furthermore, studies confirmed the validity of using AE for the estimation of UCS by studying micro-crack evolution [92,93], where some studies continued to extend the application of AE to shear failure analysis and gangue-based CPB [94,95]. Yin et al. [96] brought forward a new AE-based index for determining instability, and Chen et al. [97] highlighted the complementary application of AE in conjunction with UCS to depict the mechanical behavior of CPB. Hence, from several studies, it can be concluded that AE monitoring is a viable, online, and non-invasive technique for determining the strength, deformation, and failure of CPB or other cemented backfill with minimum dependence on destructive testing.

## 5. Comparative and Meta-Analysis of NDT Methods for UCS Prediction

**Figure 11:**
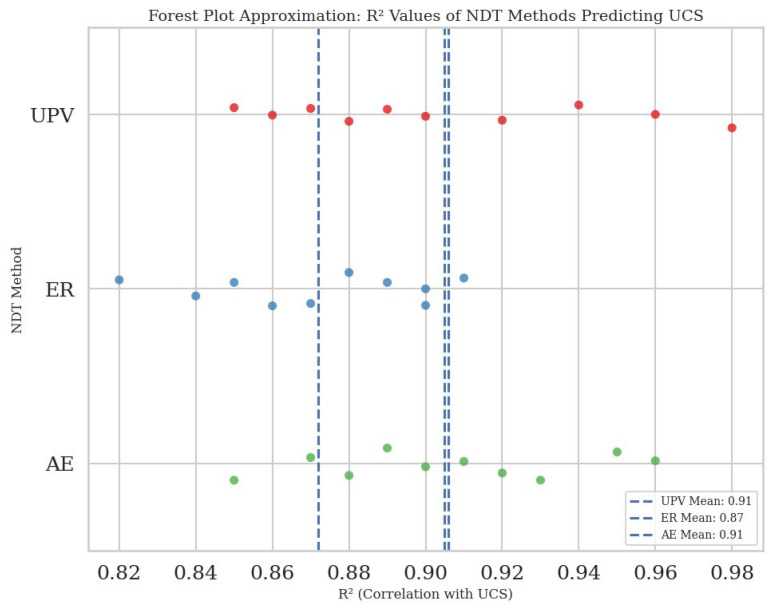
Forest plot of R2 values between NDT methods and UCS across 30 studies. This figure compares the R² values of NDT methods: UPV (green), AE (red), and ER (blue) in predicting UCS. Each point represents a study’s R², with dashed lines showing the mean R² for each method. AE shows the highest average R² value among the methods.

To quantitatively compare the performance of NDT methods in predicting the UCS of CPB, a meta-analysis was conducted using data from 30 peer-reviewed studies. The correlation coefficients (R^2^) between each method and UCS were extracted then visualized through a forest plot as shown in Figure 11.

The results indicate that the UPV and AE methods are very dependable and consistent. They have high average R^2^ values of 0.895 and 0.896, meaning they are quite accurate. These methods show little variation across different studies, which makes them robust for predicting UCS with various CPB mixes. Notably, UCS thresholds are important in high-stress environments (e.g., deep mines with σ_3_ > 5 MPa) or weak host rocks like coal where CPB must prevent roof collapse or pillar failure. Tailings composition (sulfidic vs. siliceous) further dictates UCS targets, underscoring the need for reliable NDT correlations under diverse geotechnical conditions. On the other hand, the ER method has a slightly lower average R^2^ of 0.872 and displays more variation, with R^2^ values ranging from 0.82 to 0.91. This is mostly because ER is sensitive to external conditions like moisture and temperature.

These findings suggest that all three methods are effective for assessing CPB strength without causing damage. However, UPV and AE are particularly reliable and practical in different environments. AE is excellent for detecting early signs of cracking and failure, while UPV is best for monitoring hydration and material becoming denser. The variability in ER performance highlights the importance of controlling environmental factors when using this method.

The strong correlation between these methods supports the use of hybrid systems. Combining UPV with AE or ER could improve real-time prediction, early warnings, and monitoring the health of structures in mining areas. To compare these non-destructive testing (NDT) methods, we examined correlation coefficients, error margins, and sensitivity ranges from various studies. This section provides numerical benchmarks for UPV, ER, and AE in predicting UCS, offering a more data-focused evaluation based on prior findings from sources like [24,74], and [92].

**Figure 12:**
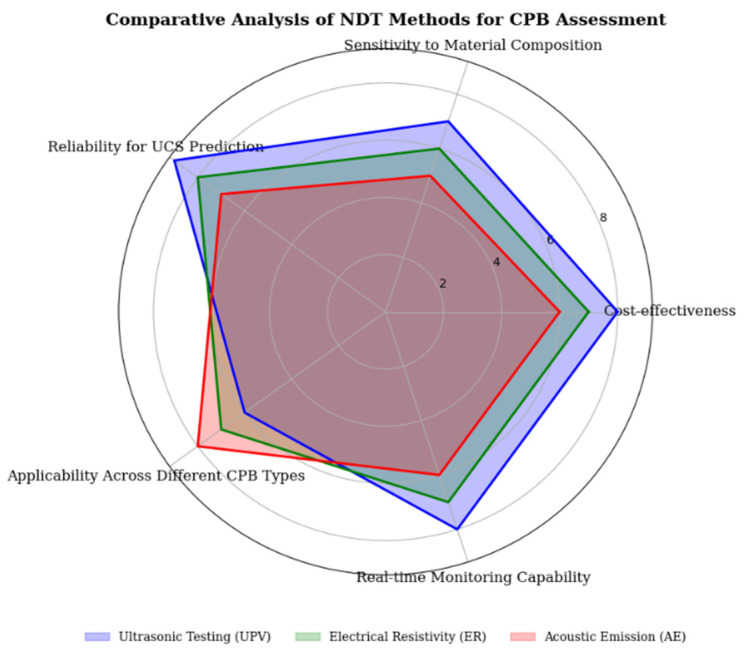
Radar Chart showing the comparative performance of UPV, ER, and AE across five critical parameters. Higher values indicate better performance in each category. Data extracted from multiple case studies (2020–2024) assessing NDT methods for CPB evaluation.

A systematic comparison of NDT methods for CPB assessment is provided in Table 6 above; however, the information is represented visually in Figure 12 using Radar chart which makes the information easily understandable and easier to analyze. It can be used effectively to compare UPV, ER and AE against a number of critical factors and show their strengths and weaknesses. This graphical approach is a comprehensive comparison tool that can be used to easily determine the most appropriate NDT method for a given application.

The UPV method has a high correlation with UCS (R^2^ = 0.85–0.95) and is therefore a very accurate non-destructive tool for estimating strength. However, the ±5% error margin, which is caused by voids, density variations, and material heterogeneity, needs to be calibrated for different CPB mixtures [41,72]. In a similar manner, ER testing is a moderate to strong UCS correlation (R^2^ = 0.78–0.92) and is suitable for hydration and microstructural monitoring [81]. However, accuracy is ±10% and it is sensitive to moisture, which means that it needs to be applied in controlled conditions or with correction factors [86]. Acoustic Emission (AE) monitoring, which correlates well with UCS (R^2^ = 0.80–0.96), can detect 85–96% of crack events before failure, providing up to 48 h of predictive lead time [92,94]. However, AE accuracy depends on sensor placement, signal noise filtering, and crack initiation conditions, requiring advanced processing techniques for enhanced reliability.

The fundamental differences in performance are from their underlying physics: UPV measures wave velocity through solids, ER tracks ionic conductivity in pore solutions, and AE records stress induced acoustic waves. The strengths of these techniques are complementary, and it is conceivable that hybrid methods that incorporate aspects of UPV, ER, and AE could represent a more holistic framework for the assessment of CPB.

## 6. Simulation-Based Validation of a Hybrid NDT Approach

To support the comparative meta-analysis of existing NDT techniques, a simulation-based assessment was conducted to evaluate the predictive strength of a hybrid NDT model that integrates UPV, AE and ER for UCS estimation. Due to the scarcity of publicly available datasets containing all three NDT parameters together, a synthetic dataset had to be developed. This dataset is like the real world in terms of material conditions and the way measurements can vary, providing a controlled environment to explore the behavior of CPB under hybrid NDT monitoring.

### 6.1. Synthetic Data Generation

A total of 100 synthetic samples were created using possible value ranges found in the literature, as projected in Table 7. The UPV values were designed with a normal distribution, averaging 3.2 km/s with a small variation in ±0.3. This represents how sound waves typically move through compacted CPB. AE values were generated using a gamma distribution to reflect the skewed nature of acoustic emissions during crack initiation and progression. ER values were developed using a uniform distribution, meaning they were evenly distributed between 50 and 120 ohm-m. This range matches the differences in moisture and small pores in the materials used to fill backfilled spaces.

The UCS is a simple linear equation made with three predictors, as shown in Equation (1). To mimic the unpredictability of real-world situations, it includes random variations to capture the uncertainties we often experience:

UCS = 10 ⋅ UPV + 0.8 ⋅ AE − 0.05 ⋅ ER + ε(1)

where ε is a Gaussian noise term with mean 0 and standard deviation 5 MPa.

### 6.2. Model Training and Evaluation

Models were trained using Python’s scikit-learn (v1.2) with an 80/20 split, balancing computational efficiency and robust validation (sufficient test samples). This ratio aligns with ML best practices for small-to-medium datasets [3,36], avoiding overfitting while preserving statistical power. Linear regression (LR) and random forest (RF) models were selected in order to evaluate how well the models works. These models used three input features—UPV, AE, and ER—to predict UCS, the target variable. We split the dataset, using 80% for training the models and 20% for testing to make sure the results are reliable. The performance results for these models can be found in Table 8, which provides a clear comparison of how each model did in making predictions.

Table 8’s results show that LR model consistently outperforms RF model when using single-input features especially with AE, while RF struggles to capture patterns with limited inputs. However, RF performance improves with hybrid inputs, particularly with the full combination (UPV + AE + ER), where it achieves its best R^2^ of 0.320. Although LR still shows slightly higher accuracy overall, the results highlight RF potential in capturing complex, non-linear relationships when multiple NDT features are integrated.

To maintain clarity and avoid redundancy in figure presentation, only the best-performing hybrid configuration is visualized. Among the three possible two-feature combinations (UPV + AE, UPV + ER, AE + ER), the UPV + AE model demonstrated the highest predictive performance with respect to R^2^ and mean absolute error (MAE). As such, it serves as a representative case to illustrate the improvement achievable through partial feature integration, while ensuring the manuscript remains concise and focused.

**Figure 13:**
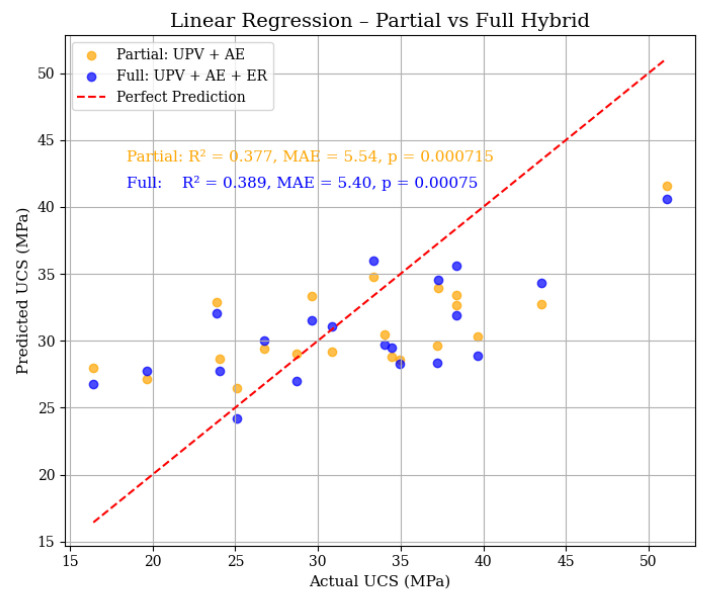
Actual versus predicted UCS using LR models trained on partial (UPV + AE) and full (UPV + AE + ER) hybrid inputs. The red dashed line represents perfect prediction (y = x). Annotated within the plot are the R^2^, MAE, and *p*-values from an auxiliary LR analysis confirming statistical significance. The inclusion of ER improves prediction accuracy modestly.

As shown in Figure 13, both partial and full models demonstrate moderate alignment with actual UCS, but the full hybrid model (blue markers) yields a marginal improvement over the partial hybrid model (orange markers). Specifically, the full hybrid configuration achieves an R^2^ of 0.389 and MAE of 5.40 MPa, and a significant *p*-value of 0.00075, compared to the partial model R^2^ of 0.377 and MAE of 5.54 MPa, and *p*-value of 0.000715. Although the improvement might seem small, it shows the benefit of including ER in predicting UCS. This is important when small improvements can lead to more reliable assessments of structures. Such enhancements are especially valuable for CPB and similar materials. The results demonstrate that even with straightforward methods, using various types of data can improve the accuracy of strength evaluations.

**Figure 14:**
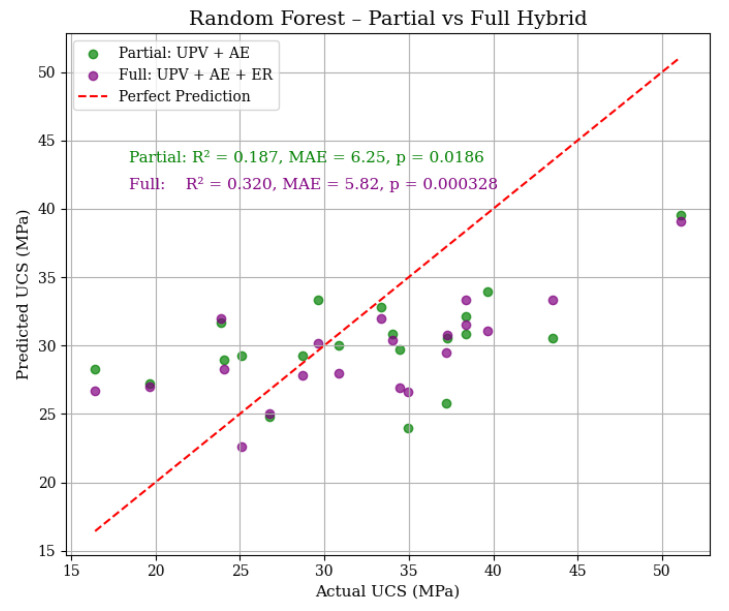
Actual versus predicted UCS using RF models with partial (UPV + AE) and full (UPV + AE + ER) hybrid inputs. The red dashed line indicates the ideal prediction line. R^2^, MAE, and *p*-values are annotated within the plot, showing that the full hybrid model significantly improves prediction accuracy compared to the partial hybrid configuration. These results highlight the advantage of incorporating multiple NDT parameters for more reliable UCS estimation.

As shown in Figure 14, the full hybrid model (purple markers) clearly shows better clustering along the perfect prediction line, with an R^2^ of 0.320 and MAE of 5.82 MPa, and a significant *p*-value of 0.000328, compared to the partial model R^2^ of 0.187 and MAE of 6.25 MPa, and *p*-value of 0.0186. This performance improvement confirms the value of incorporating ER alongside UPV and AE, especially in ML frameworks capable of capturing non-linear feature interactions. Unlike LR, where adding ER had marginal impact, RF capitalizes more effectively on the diversity of input data. These findings support the conclusion that a full hybrid NDT approach enhances model generalization and prediction accuracy, offering practical benefits for reliable UCS assessment in cement-based materials.

### 6.3. Residual and Feature Analysis

**Figure 15:**
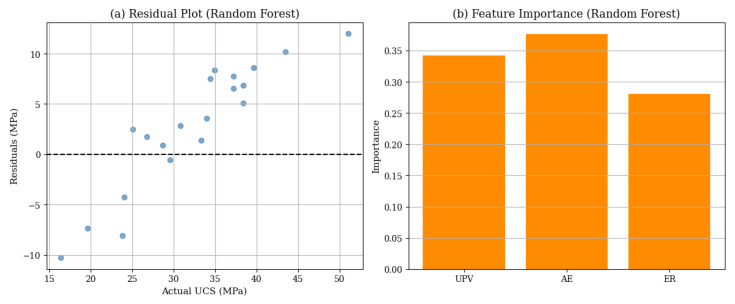
RF model results using hybrid NDT inputs (UPV, AE, ER), with (**a**) showing the residual plot and moderate prediction errors around the zero line, and (**b**) showing the feature importance plot identifying AE and UPV as dominant predictors of UCS.

Figure 15 offers a deeper understanding of how the RF model operates with hybrid NDT inputs. The residual plot (a) shows that prediction errors are spread around the zero line, indicating that the model performs well without obvious bias or underfitting issues. The feature importance plot (b), reveals that AE and UPV are the most significant for the model predictions, while ER contributes less but is still helpful. This means that combining all three NDT methods improves the model’s accuracy, but AE and UPV have more influence in predicting UCS. These perceptions confirm the usefulness of the hybrid method and help professionals decide which NDT variables are most important when data is limited.

### 6.4. Discussion and Broader Relevance

The simulation demonstrates that combining different NDT methods, such as UPV, AE, and ER results in more accurate predictions than relying on any single method. This combined approach is effective not only for CPB conditions but also for other cement and pavement materials. UPV and AE are commonly used in road diagnostics to detect issues like compaction problems and fatigue cracking. ER helps identify moisture-related damage. Thus, the hybrid NDT model offers a reliable way to assess the strength of structures without causing harm. It can be applied in smart road systems, quality assurance and asset management, making it a valuable tool for future infrastructure projects. To enhance this model further, future work should involve testing it with real-world data to ensure its effectiveness and applicability.

## 7. Conclusions

This study reviewed and benchmarked the effectiveness of UPV, AE, and ER as NDT for estimating the compressive strength of CPB and similar cementitious systems. Thirty peer-reviewed studies were analyzed, selecting ten for each method and conducting meta-analyses to quantitatively compare their performance using reported R^2^ values. The findings reveal that UPV proved to be the most dependable for predicting strength in different mixtures and at different curing times. AE and ER were also useful but less consistent. Each method provides unique insights into material properties, such as density, presence of cracks, and moisture content.

To better understand how a hybrid diagnostic strategy could be more effective, a synthetic simulation dataset was created using data from all three NDT techniques. Researchers developed linear regression and random forest models to predict material strength more accurately. The hybrid model which utilized the combined dataset outperformed the models that relied on just one NDT input. It achieved an R^2^ value of 0.389 and MAE of 5.40 MPa. Analyzing the errors showed that the hybrid model was better at reducing both bias and prediction mistakes.

Additionally, when looking at which features were most important, it became clear that UPV, AE, and ER each played a key role in enhancing the model performance. Although this simulation was designed around CPB conditions, the proposed hybrid framework is transferable to pavement and asphalt materials, where similar NDT techniques are used to examine compaction quality, fatigue cracking and subsurface moisture damage. The model is useful for both underground and surface materials, making it a valuable tool for sustainable and non-invasive checking in infrastructure projects.

This study presents a two-pronged approach combining systematic review and simulation modeling to link current NDT methods with ways to predict future conditions. Demonstrating the hybrid NDT approach is an important step toward using advanced tools for quality checks, maintenance planning, and developing modern infrastructure systems. Future work should focus on gathering real-world data for this method, testing the models in actual field conditions and exploring how these methods can work together with new sensors and IoT technologies for ongoing monitoring.

## Figures and Tables

**Table 1:** Comparison of destructive testing methods for CPB: key characteristics, measured properties, and applications of UCS, Triaxial and Brazilian tests.

Test	Load Type	Measured Property	Confining Pressure	Typical Use Case
UCS	Uniaxial	Compressive strength (σ_e_)	None (σ_2_ = σ_3_ = 0)	Basic strength screening
Triaxial	Multiaxial	Shear strength (c, φ)	Controlled (σ_2_ = σ_3_ ≠ 0)	Deep mining, slope stability
Brazilian	Diametrical	Tensile strength (σ_t_)	None	Crack resistance, brittle materials

**Table 2:** A detailed comparison of DT and NDT methods focusing on how they perform in terms of resources conservation, material integrity preservation and cost-effectiveness.

Feature	Destructive Testing	Non-Destructive Testing
Sample Integrity	Destroys samples	Retains specimens for further use
Testing Time	Time-consuming	Faster, real-time assessment
Cost	High due to multiple samples	Lower operational costs
Real-Time Monitoring	Not possible	Possible with UPV, ER, and AE
Field Application	Limited to controlled labs	Suitable for both lab and field
Data Collection	Single-point data (after failure)	Continuous monitoring possible

**Table 3:** Comparison of UPV and UCS correlation studies, showing the effectiveness of UPV as a non-destructive alternative for UCS prediction across different material types.

Study	Focus Area	Methodology	Key Findings	Conclusion
Yilmaz & Ercikdi [24]	Correlation of UCS and UPV in CPB with different tailings	180 CPB samples, UCS and UPV testing over 7–56 days, regression analysis	Strong correlation (0.79–0.95) between UCS and UPV, confirming UPV as a UCS predictor	UPV provides a reliable, non-destructive alternative for UCS estimation in CPB
Wu et al. [74]	Mechanical performance of cemented gangue backfills using UCS and UPV	60 CGB samples, UCS and UPV testing over 3–28 days, exponential relationship analysis	High correlation (0.959) between UCS and UPV, with strength growth over time	UPV effectively predicts UCS in cemented gangue backfill without sample damage
Xu et al. [75]	Strength and ultrasonic properties of foam-cemented paste backfill	FCPB samples with varying vesicant-binder ratios, UCS and UPV correlation	Linear correlation between UCS and UPV, improving accuracy with lower vesicant ratios	UPV serves as a useful, non-destructive UCS estimator in foam-cemented paste backfill
Mendes et al. [76]	Use of UPV as a performance parameter for concrete mixture design	68 concrete mixtures, UCS and UPV tests at 28 and 91 days, silica fume impact analysis	UPV effectively predicts concrete strength except for high SF content mixtures	UPV is a viable non-destructive indicator of concrete performance and strength
Jiang et al. [16]	UPV and UCS relationship in alkali-activated slag (AAS)-based CPB	Various CPB mixtures, UCS and UPV testing at different curing ages, regression modeling	Exponential correlation (R^2^ > 0.94) between UCS and UPV in AAS-CPB	UPV provides cost-effective, efficient UCS predictions for AAS-CPB
Nugroho et al. [77]	Impact of mortar mixture variations on compressive strength and UPV	Examining fine aggregate-cement ratios, UCS and UPV logarithmic correlation analysis	UPV and UCS follow a logarithmic trend, inversely related to porosity	UPV offers real-time assessment of mortar properties for mixture optimization
Hong et al. [78]	Compressive strength and UPV correlation in silica fume-blended cement mortars	ISO 679 standard [27]-based testing, SF influence on UCS and UPV correlation	UPV and UCS show an exponential relationship, influenced by SF replacement rate	UPV measurement serves as a practical alternative to destructive UCS testing
Sari et al. [13]	Silica fume (SF)-replacement’s impact on UPV and compressive strength in cement mortar	Different SF replacement rates in cement mortar, UCS and UPV correlation analysis	SF substitution significantly influences UCS-UPV correlation, with minor effects on overall trend	UPV remains a strong indicator of UCS across different SF replacement rates
Aslam et al. [79]	Mechanical and durability properties of sustainable lightweight concrete with WPA	Concrete with WPA (20–100%), UCS and UPV polynomial regression analysis	UPV decreases with higher WPA, affecting UCS due to increased porosity and reduced density	UPV can effectively assess mechanical integrity of sustainable lightweight concrete
Kowsalya et al. [53]	Predicting compressive strength of Fly Ash Cenosphere (FAC) concrete using UPV	FAC concrete, IS 516:2018 [51] and IS 13311-1992 standard [52]-based UCS and UPV testing	UPV exhibits strong exponential correlation (0.91–0.99) with UCS, confirming prediction reliability	UPV provides a non-destructive means of evaluating the compressive strength of FAC concrete

**Table 4:** Comparison of ER and UCS correlation studies, highlighting the sustainability benefits of ER in waste reduction, real-time monitoring, and cost-effective testing.

Study	Focus Area	Methodology	Key Findings	Conclusion
Xu et al. [80]	Use of ER in CPB geotechnical evaluation	Wenner Four-Electrode method, UCS testing, ASTM G57-06 standards [17]	ER correlated significantly with UCS; rapid early strength gain noted.	ER testing is a cost-effective, time-efficient alternative to UCS.
Xu et al. [60]	Predicting UCS through ER in CPB hydration process	CPB samples with varying cement ratios tested for ER and UCS correlation	ER effectively predicted UCS development throughout hydration.	ER can reliably monitor hydration progress and strength in CPB.
Liu et al. [81]	Correlation between ER, hydration heat, and UCS	NER and microcalorimetry for resistivity, UCS testing for mechanical assessment	Strong correlation found between ER, hydration heat, and UCS.	Resistivity serves as a strong predictive indicator of UCS and hydration.
He et al. [82]	Mechanical behavior of coal waste rock backfills	Compression tests on coal waste rock backfill, ER measured via two-phase and four-phase methods	ER method effectively monitored deformation characteristics of waste rock backfill.	ER testing is a practical tool for monitoring backfill mechanical behavior.
Wu et al. [25]	Comparing ER and AE for UCS alternative testing	Analyzed ultrasonic waveforms, electrical conductivity, hydration heat, and ER-UCS correlation	ER and AE provided alternative strength evaluation methods to UCS.	ER complements AE as a real-time UCS testing alternative.
Zhu et al. [83]	Evaluating ICPB properties using ER	Investigated microstructure and hydration using NMR, SEM-EDS, and FFRC tests	ER correlated with UCS, capturing hydration and porosity changes.	ER effectively evaluates ICPB properties, including hydration and strength.
Lee et al. [84]	Application of ER in cement-based materials	Examined hydration, curing time, water-solid ratios’ impact on ER in cement pastes	ER values increased with hydration and material density, indicating strength evolution.	ER is a viable tool for assessing early-age cement-based materials.
Zhang et al. [85]	ER-UCS relationship under varying curing temperatures	CPB specimens cured at 20–60 °C to study hydration effects on ER and UCS	Optimal strength achieved at 40 °C; ER tracked hydration efficiency.	ER testing provides real-time insights into UCS evolution in CPB.
Wan et al. [86]	Effect of calcium nitrite on ER-UCS correlation	Studied calcium nitrite impact on hydration and microstructure using ER and UCS tests	Higher ER values indicated denser structure and stronger UCS correlation.	ER reliably predicts UCS, supporting improved mix design strategies.
Gholampoor et al. [87]	ER as a tool for early-stage quality of stabilized DS	Monitored hydration and ion concentration using ER, compared with UCS at 28 days	ER provided early prediction of UCS, enabling non-destructive quality assessment.	ER testing enables rapid and non-destructive quality assessment of DS.

**Table 5:** Summary of AE monitoring studies correlating AE signals with UCS in CPB and other materials, highlighting AE as a reliable non-destructive method for tracking damage evolution and material failure.

Study	Focus Area	Methodology	Key Findings	Conclusion
Cao et al. [88]	AE monitoring for waste rock CPB under triaxial compression	Triaxial compression tests with AE monitoring; stress–strain behavior analysis	AE activity mirrors UCS failure stages; prepeak quiescence and postpeak calm observed	AE serves as a reliable, real-time alternative to UCS for assessing mechanical strength
Wu et al. [89]	AE monitoring as an alternative to UCS for CPB	Uniaxial compression tests with AE21C system; crack activity analysis	AE counts peak at stress maximum; AE activity increases with cement content	AE monitoring effectively captures stress–strain behavior in CPB
Wang et al. [90]	Mechanical behavior and AE properties of CPB	PCI-2 AE system with six sensors; stress–strain monitoring during UCS tests	AE ringing count increases with load; AE signals correlate with damage evolution	AE provides insights into crack propagation and structural integrity
Zhao et al. [91]	AE analysis of cemented tantalum-niobium tailings backfills	UCS and Brazilian splitting tests; AE signal analysis of stress response	AE peak timing varies with lime-sand ratio; microcrack dynamics analyzed	AE correlates with stress-induced damage and predicts UCS values
Zhou et al. [92]	AE for assessing UCS and damage evolution in CPB	UCS tests and AE signal tracking; event rate and energy rate correlation	Strong correlation between AE properties and UCS failure stages	AE enables real-time assessment of CPB failure characteristics
Qiu et al. [93]	AE characteristics in cemented rock-tailings backfill	UCS and AE monitoring of cemented rock-tailings backfill with varying compositions	AE parameters like ringing count and cumulative hits track UCS evolution	AE analysis aids in optimizing waste rock content in cemented backfill
Chen et al. [94]	AE monitoring under variable angle shear tests	AE monitoring under shear tests at 30°, 45°, and 60° angles	AE energy evolution follows shear angles; stepwise failure at 60°	AE monitoring complements UCS in evaluating shear failure mechanics
Sun et al. [95]	AE for Gangue and high water-CPB mechanical assessment	AE monitoring of stress–strain behavior during UCS tests	AE signals correspond to internal damage progression in CPB	AE tracking enhances UCS predictions in gangue-based CPB
Yin et al. [96]	AE application in CPB-rock combination failure assessment	AE monitoring with rise time, amplitude, average frequency value analysis	AE b-value and Dr indicator developed for failure prediction	AE b-value and Dr offer robust predictive tools for instability
Chen et al. [97]	AE integration with UCS testing for CPB analysis	AE ringing count correlation with UCS stress levels in CPB	AE and UCS tests complement each other for material stability assessment	AE supports non-destructive testing and UCS measurement integration

**Table 6:** Summary of meta-analysis results for NDT methods (UPV, ER, and AE) based on R^2^ correlation with UCS, showing the predictive strength and variability of each method.

NDT Method	Mean (R^2^)	Range	Insight
UPV	0.895	0.85–0.95	Strong and consistent predictor of UCS; minimal spread shows reliability.
ER	0.872	0.78–0.92	Slightly lower mean R^2^ with higher variability due to moisture sensitivity.
AE	0.896	0.80–0.96	Comparable to UPV in strength prediction; shows tight clustering, excellent for crack progression detection.

**Table 7:** A summary of the synthetic data generated for the studies (see Supplementary Materials for full dataset).

UPV (km/s)	AE (Units)	ER (ohm-m)	UCS (MPa)
3.149	2.221	90.44	29.638
3.335	7.175	112.79	42.654
3.536	3.273	102.97	37.580
……	……	…….	…....

**Table 8:** LR and RF model performance across different NDT input configurations for UCS prediction.

Model Input	R^2^ (LR)	MAE (LR)	R^2^ (RF)	MAE (RF)
UPV only	0.148	6.32	0.063	5.99
AE only	0.291	5.89	−0.083	7.07
ER only	−0.035	6.60	0.020	6.13
UPV + AE	0.377	5.54	0.187	6.25
UPV + ER	0.149	6.22	0.069	6.23
AE + ER	0.318	5.49	0.101	6.63
UPV + AE + ER	0.389	5.40	0.320	5.82

## Data Availability

The original contributions presented in this study are included in the article. Further inquiries can be directed to the corresponding author.

## Supplementary Materials

The following supporting information can be downloaded at: https://www.mdpi.com/article/10.3390/ma18122888/s1, Synthetic NDT and UCS Data.

## Abbreviations

AAS	Alkali-Activated Slag
AE	Acoustic Emission
CPB	Cemented Paste Backfill
ER	Electrical Resistivity
LR	Linear Regression
NDT	Non-Destructive Testing
RF	Random Forest
R^2^	Coefficient of Determination
UCS	Uniaxial Compressive Strength/Unconfined Compressive Strength
UPV	Ultrasonic Pulse Velocity
DT	Destructive Testing
MAE	Mean Absolute Error
ASTM	American Society for Testing and Materials
ISO	International Organization for Standardization
SF	Silica Fume
WPA	Waste Pumice Aggregate
FAC	Fly Ash Cenosphere
NER	Normalized Electrical Resistivity
ICPB	Improved Cemented Paste Backfill
DS	Dredged Sediment
IoT	Internet of Things
