# Supplementary material for: Comparative and Meta-Analysis Evaluation of Non-Destructive Testing Methods for Strength Assessment of Cemented Paste Backfill: Implications for Sustainable Pavement and Concrete Materials
Source: Materials (Basel). 2025 Jun 18;18(12):2888. doi: 10.3390/ma18122888 (PMC12195205; doi:10.3390/ma18122888)
Supplement: Supplementary file 1 [file materials-18-02888-s001.zip › materials-3668610-supplementary.pdf]

Synthetic NDT and UCS Data

UPV (km/s)	AE (units)	ER (ohm-m)	UCS (MPa)
3.349	0.852	76.223	26.768
3.159	2.361	93.810	27.716
3.394	2.934	85.220	33.584
3.657	4.489	109.954	42.040
3.130	4.044	96.109	34.015
3.130	3.145	61.405	29.943
3.674	0.428	54.940	34.238
3.430	3.265	94.969	27.153
3.059	3.491	51.856	30.699
3.363	14.596	91.004	39.311
3.061	2.861	115.816	28.721
3.060	4.174	90.283	25.292
3.273	7.240	77.172	37.256
2.626	5.676	95.030	33.713
2.683	8.426	82.078	28.918
3.031	0.866	88.193	28.605
2.896	1.374	115.903	27.716
3.294	2.075	77.027	28.745
2.928	0.719	117.283	25.107
2.776	3.514	113.375	24.968
3.640	1.478	63.705	34.883
3.132	9.150	54.855	32.035
3.220	1.692	57.054	30.826
2.773	2.569	51.276	29.707
3.037	5.906	56.611	39.517
3.233	1.058	97.810	33.085
2.855	0.667	54.983	37.098
3.313	3.833	72.328	28.740
3.020	4.050	109.141	32.343
3.112	5.787	51.629	34.090
3.019	4.871	107.013	39.690
3.756	4.160	69.730	33.357
3.196	1.868	58.272	26.342
2.883	3.970	98.772	24.067
3.447	4.149	94.026	22.466
2.834	1.808	111.423	21.584
3.263	10.844	101.455	32.433
2.612	4.713	106.244	25.331
2.802	1.397	69.742	27.355
3.259	5.807	62.421	43.496
3.422	6.491	102.543	39.033
3.251	4.517	106.478	27.919
3.165	2.739	119.335	23.385
3.110	1.741	78.883	31.005
2.756	3.138	76.041	19.672
2.984	4.294	104.349	37.216
3.062	3.367	73.856	35.516
3.517	8.671	115.153	34.005
3.303	5.224	110.089	23.140
2.671	1.574	80.030	30.738

3.297	1.262	102.561	28.281
3.084	4.741	102.818	35.685
2.997	2.789	57.219	21.367
3.384	5.538	113.179	29.609
3.509	4.711	85.368	34.619
3.479	3.149	107.852	32.155
2.948	1.590	72.403	24.884
3.107	0.752	112.687	29.154
3.299	3.917	77.244	26.927
3.493	1.041	50.759	32.509
3.056	1.532	113.377	26.721
3.144	3.746	56.390	34.193
2.868	3.486	72.352	31.410
2.841	1.167	116.504	17.897
3.444	6.985	116.543	26.528
3.607	6.862	90.141	43.439
3.178	4.848	94.229	32.612
3.501	4.844	81.391	31.074
3.308	4.848	70.525	41.193
3.006	6.453	73.007	32.155
3.308	5.314	97.076	38.378
3.661	1.710	102.666	33.186
3.189	2.758	105.411	39.133
3.669	13.593	105.273	51.081
2.414	0.463	56.384	20.447
3.447	2.257	84.609	36.899
3.226	7.010	54.029	38.394
3.110	1.807	88.467	34.968
3.228	5.414	80.907	27.736
2.604	3.452	112.139	26.622
3.134	1.918	74.564	34.439
3.307	0.362	58.195	21.658
3.643	3.838	60.009	30.588
3.045	1.677	103.306	16.426
2.957	3.046	93.275	26.000
3.049	4.815	57.079	35.080
3.475	6.106	55.887	44.348
3.299	2.251	99.068	30.204
3.041	1.915	55.093	37.331
3.354	1.023	107.530	22.081
3.229	6.309	99.437	23.850
3.491	12.363	55.694	41.734
2.989	6.773	55.939	34.436
3.102	7.791	119.065	31.133
3.082	1.820	76.199	18.132
2.761	4.616	75.945	27.059
3.289	5.760	106.896	25.629
3.278	1.326	116.307	31.377
3.202	9.574	119.020	35.557
3.130	0.836	102.736	22.129
